# Supplementary figures and images for: A comparison of blood gases, biochemistry, and hematology to ecomorphology in a health assessment of pinfish (Lagodon rhomboides)
Source: PeerJ. 2016 Aug 9;4:e2262. doi: 10.7717/peerj.2262 (PMC4991879; doi:10.7717/peerj.2262)

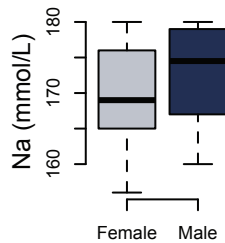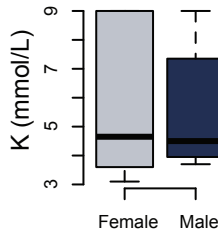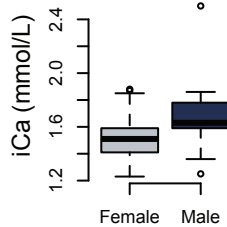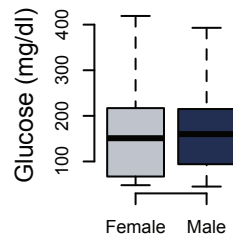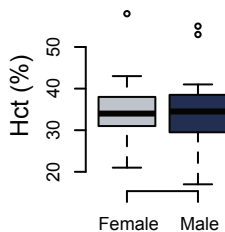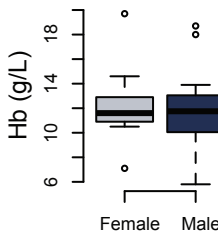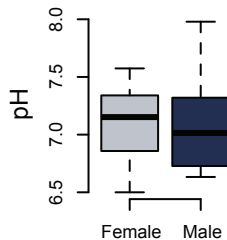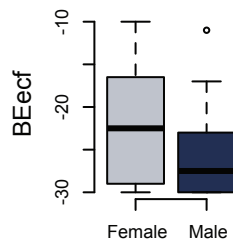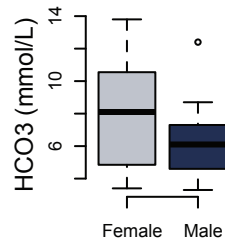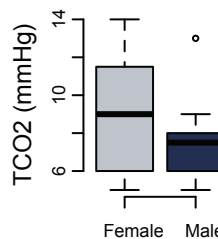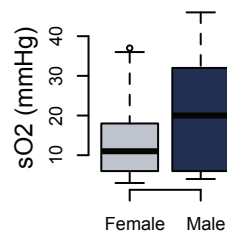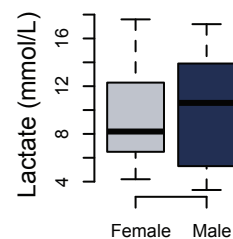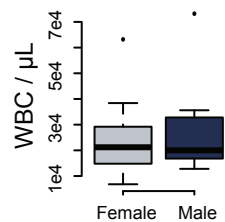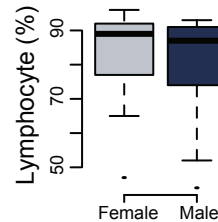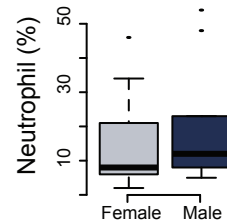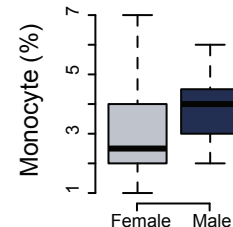

Supplement: Figure S1 — Box plots comparing the quartiles of measured blood parameters between male and female pinfish. [file peerj-04-2262-s002.pdf]

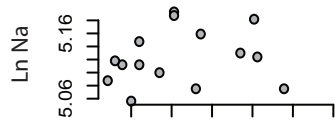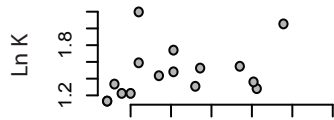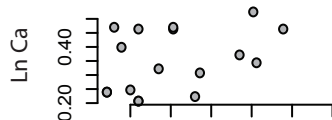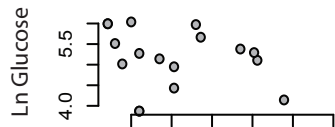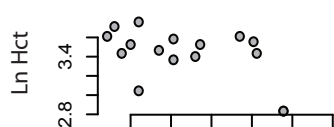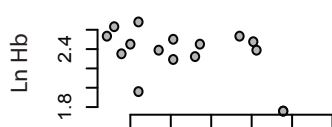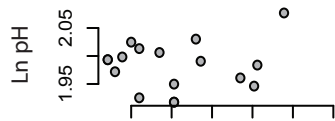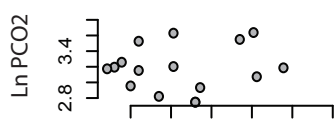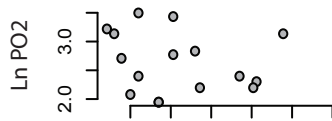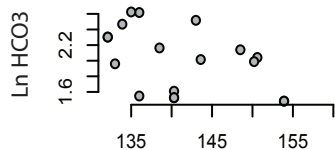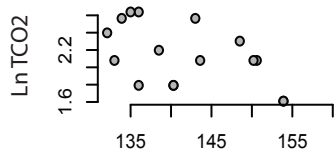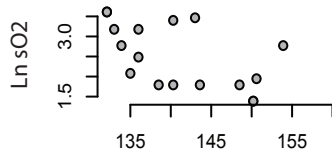

Lower Jaw Angle

Lower Jaw Angle

Lower Jaw Angle

Supplement: Figure S2 — Scatterplots comparing the distribution of hematological and blood chemistry values (log scale) to jaw angle measurements. [file peerj-04-2262-s003.pdf]
