# Supplementary material for: A comparison of blood gases, biochemistry, and hematology to ecomorphology in a health assessment of pinfish (Lagodon rhomboides)
Source: PeerJ. 2016 Aug 9;4:e2262. doi: 10.7717/peerj.2262 (PMC4991879; doi:10.7717/peerj.2262)
Supplement: Table S1 — The effect of body size on jaw morphology values. Coefficients and 95% confidence values for the SMA regression model testing for a possible effect of body size on each trait or significantly different effects between males and females. [file peerj-04-2262-s004.docx]

| \| **Table S1**: The effect of body size on jaw morphology values. Coefficients and 95% confidence values for the SMA regression model testing for a possible effect of body size on each trait or significantly different effects between males and females. \| \| --- \| | | | | | | | |
| --- | --- | --- | --- | --- | --- | --- | --- | --- |
|  |  | Upper Jaw Protrusion | Lower Jaw Protrusion | Gape | Lower Jaw Angle | Middle Jaw Angle | Upper Jaw Angle |
| Female | Elevation | -4.29 (-8.42, -0.16) | -4.69 (-8.17, -1.21) | -3.74 (-6.75, 0.73) | 7.33 (5.85, 8.81) | 0.008 (-2.61, 2.63) | 6.35 (5.49, 7.21) |
|  | Slope | 1.29 (0.70, 2.38) | 1.45 (0.91, 2.32) | 1.22 (0.76, 1.98) | -0.48 (0.087, -0.26) | 0.80 (0.43, 1.50) | -0.32 (-0.54, -0.19) |
| R-squared / P |  | **0.49 / 1 / 1.6e-2** | **0.64 / 3.0e-3** | 0.26 / 0.24 | 0.002 / 0.98 | 0.03 / 0.98 | 0.13 / 0.51 |
| Male | Elevation | -4.24 (-6.20, -2.27) | -3.78 (-6.34, -1.21) | -2.55 (-4.16, -0.94) | 2.83 (1.45, 4.2) | 1.05 (-0.45, 2.57) | 3.74 (3.00, 4.49) |
|  | Slope | 1.30 (0.96, 1.76) | 1.28 (0.86, 1.90) | 1.00 (0.72, 1.37) | 0.42 (0.23, 0.78) | 0.58 (0.35, 0.96) | 0.20 (0.10, 0.40) |
| R-squared / P |  | **0.79 / 8.85-05** | **0.81 / 5.82e-5** | **0.78 / 1 / 8.85e-5** | 7.4e-4 / 0.92 | 0.12 / 0.87 | 0.069 / 0.87 |
| H0 : slopes are equal | Likelihood Ratio Test / DF / P | 1.27e-3 / 1 / 1 | 0.1734 / 1 / 1 | 0.535 / 1 / 1 | 0.088 / 1 / 1 | 0.69 / 1 / 1 | 1.097 / 1 / 1 |
