# Supplementary material for: A comparison of blood gases, biochemistry, and hematology to ecomorphology in a health assessment of pinfish (Lagodon rhomboides)
Source: PeerJ. 2016 Aug 9;4:e2262. doi: 10.7717/peerj.2262 (PMC4991879; doi:10.7717/peerj.2262)
Supplement: Table S2 — The effect of body size on hematological values. Coefficients and 95% confidence values for the SMA regression model testing for a possible effect of body size on each trait or significantly different effects between males and females. PCV, Packed cell volume; WBC, White blood cell count. [file peerj-04-2262-s005.docx]

| **Table S2**: The effect of body size on hematological values. Coefficients and 95% confidence values for the SMA regression model testing for a possible effect of body size on each trait or significantly different effects between males and females. PCV = Packed cell volume, WBC = White blood cell count. | | | | | | | | | |
| --- | --- | --- | --- | --- | --- | --- | --- | --- | --- |
|  |  | Sodium | Potassium | Calcium | Glucose | Hematocrit | Hemoglobin | pH | PCO_2_ |
| Female | Elevation | 6.97 (6.11, 7.82) | -20.42 (-34.14, -6.70) | -4.93 (-7.93, -1.94) | 34.16 (17.95, 50.38) | -3.98 (-9.65, -1.69) | -4.80 (-10.27, 0.67) | 3.82 (2.85, 4.78) | -13.41 (-21.73, -5.09) |
|  | Slope | -0.37 (-0.59, -0.24) | 4.47 (2.49, 8.04) | 1.09 (0.64, 1.86) | -5.98 (-10.13, -3.53) | 1.53 (0.76, 3.06) | 1.46 (0.74, 2.95) | -0.38 (-0.62, -0.23) | 3.42 (2.12, 5.51) |
| R-squared / P |  | 0.37/ 0.16 | 4.02e-3 /1 | 3.30e-3/1 | 1.36e-4/1 | 8.25e-3 /1 | 9.74e-3 /1 | 0.07/01 | 0.03/1 |
| Male | Elevation | 3.40 (2.31, 4.48) | -13.08 (-22.11, -4.04) | 5.48 (2.19, 8.77) | 28.51 (14.74, 42.27) | 15.40 (5.99, 24.80) | 14.24 (4.89, 23.60) | 3.92 (2.79, 5.06) | -13.95 (-23.32, -4.59) |
|  | Slope | 0.36 (0.20, 0.64) | 2.97 (1.67, 5.30) | -1.01 (-1.88, -0.54) | -4.75 (-8.29, -2.73) | -2.39 (-4.94, -1.15) | -2.37 (-4.91, -1.14) | -0.40 (-0.69, -0.23) | 3.52 (2.10, 5.89) |
| R-squared / P |  | 0.06/ 1 | 0.05/1 | 0.01/1 | 0.08/1 | 3.46e-3 /1 | 3.77e-3 /1 | 2.19e-5 /1 | 0.03/1 |
| H0 : slopes are equal | Likelihood Ratio Test / DF / P | 0.02 with 1 degree of freedom/ 1 | 1.02 with1 degree of freedom/1 | 0.03 with 1 degree of freedom/ 1 | 0.36 with 1 degree of freedom/ 1 | 0.78 with 1 degree of freedom/1 | 0.88 with 1 degree of freedom/ 1 | 0.02 with 1 degree of freedom/ 1 | 6.36e-3 with 1 degree of freedom/ 1 |
|  |  | PO_2_ | BEcf | HCO^3^ | TCO_2_ | sO_2_% | Lactate | PCV | WBC |
| Female | Elevation | -23.03 (-37.23, -8.83) | -10.39 (-17.91, -2.86) | 19.40 (8.93, 29.87) | 16.75 (7.97, 25.52) | -32.61 (-57, -8.22) | 19.51 (11.16, 27.87) | 10.09 (5.80, 14.38) | -12.19 (-27.71,3.31) |
|  | Slope | 5.18 (3.04, 8.82) | 2.73 (1.60, 4.67) | -3.54 (-6.27, -2.00) | -2.97 (-5.26, -1.68) | 7.13 (3.73, 13.66) | -3.53 (-5.62, -2.22) | -1.12 (-2.39, -0.64) | 4.5 (2.34,8.65) |
| R-squared / P |  | 0.02/1 | 0.06/1 | 0.08/1 | 0.06/1 | 0.10/1 | 0.05/1 | 0.01/1 | 0.02/1 |
| Male | Elevation | 32.71 (15.77, 49.65) | -4.72 (-9.91, 0.46) | 14.28 (6.79, 21.78) | 11.45 (5.94, 16.94) | 31 (15.89, 46.11) | -16.42 (-27.66, -5.18) | 12.80 (6.53, 19.08) | -6.21 (-16.83,4.41) |
|  | Slope | -6.11 (-10.43, -3.58) | 1.61 (0.87, 2.97) | -2.53 (-4.47, -1.43) | -1.92 (-3.33, -1.11) | -5.73 (-9.56, -3.44) | 3.78 (2.14, 6.69) | -1.81 (-3.48, -0.94) | 3.29 (1.77, 6.06) |
| R-squared / P |  | 0.13/1 | 0.05/1 | 0.04/1 | 0.03/1 | 0.50/0.32 | 0.03/1 | 0.01/1 | 0.001/1 |
| H0 : slopes are equal | Likelihood Ratio Test / DF / P | 0.20 with 1 degree of freedom/ 1 | 1.71 with 1 degree of freedom/ 1 | 0.71 with 1 degree of freedom/ 1 | 1.23 with 1 degree of freedom/ 1 | 0.3 with 1 degree of freedom/ 1 | 0.04 with 1 degree of freedom/ 1 | 0.65 with 1 degree of freedom/ 1 | 0.50 with 1 degree of freedom / 1 |
